# Supplementary material for: COZOID: contact zone identifier for visual analysis of protein-protein interactions
Source: BMC Bioinformatics. 2018 Apr 6;19:125. doi: 10.1186/s12859-018-2113-6 (PMC5889581; doi:10.1186/s12859-018-2113-6)
Supplement: Supplementary file 2 — Software build. Executable binary file of the software tool. (ZIP 118,784 kb) [file 12859_2018_2113_MOESM2_ESM.zip › cozoid/platform/docs/testng-6.5.1-javadoc.zip!/org/testng/AssertJUnit.html]

AssertJUnit (TestNG)


---


|  |  |  |
| --- | --- | --- |
| **Overview** \| **Package** \| **Class** \| **Tree** \| **Deprecated** \| **Index** \| **Help** | |  |
| **PREV CLASS**   **NEXT CLASS** | **FRAMES**    **NO FRAMES**     **All Classes** |  |
| SUMMARY: NESTED \| FIELD \| CONSTR \| METHOD | DETAIL: FIELD \| CONSTR \| METHOD |  |


---


## org.testng Class AssertJUnit

```
java.lang.Object
  org.testng.internal.junit.ArrayAsserts
      org.testng.AssertJUnit
```

---

``` public class AssertJUnit extends org.testng.internal.junit.ArrayAsserts ```

A set of assert methods. Messages are only displayed when an assert fails.
Renamed from `junit.framework.Assert`.

---

| **Constructor Summary** | |
| --- | --- |
| `protected` | `AssertJUnit()`             Protect constructor since it is a static only class |


| **Method Summary** | |
| --- | --- |
| `static void` | `assertEquals(boolean expected, boolean actual)`             Asserts that two booleans are equal. |
| `static void` | `assertEquals(byte[] expected, byte[] actual)` |
| `static void` | `assertEquals(byte expected, byte actual)`             Asserts that two bytes are equal. |
| `static void` | `assertEquals(char expected, char actual)`             Asserts that two chars are equal. |
| `static void` | `assertEquals(double expected, double actual, double delta)`             Asserts that two doubles are equal concerning a delta. |
| `static void` | `assertEquals(float expected, float actual, float delta)`             Asserts that two floats are equal concerning a delta. |
| `static void` | `assertEquals(int expected, int actual)`             Asserts that two ints are equal. |
| `static void` | `assertEquals(long expected, long actual)`             Asserts that two longs are equal. |
| `static void` | `assertEquals(java.lang.Object expected, java.lang.Object actual)`             Asserts that two objects are equal. |
| `static void` | `assertEquals(short expected, short actual)`             Asserts that two shorts are equal. |
| `static void` | `assertEquals(java.lang.String message, boolean expected, boolean actual)`             Asserts that two booleans are equal. |
| `static void` | `assertEquals(java.lang.String message, byte[] expected, byte[] actual)` |
| `static void` | `assertEquals(java.lang.String message, byte expected, byte actual)`             Asserts that two bytes are equal. |
| `static void` | `assertEquals(java.lang.String message, char expected, char actual)`             Asserts that two chars are equal. |
| `static void` | `assertEquals(java.lang.String message, double expected, double actual, double delta)`             Asserts that two doubles are equal concerning a delta. |
| `static void` | `assertEquals(java.lang.String message, float expected, float actual, float delta)`             Asserts that two floats are equal concerning a delta. |
| `static void` | `assertEquals(java.lang.String message, int expected, int actual)`             Asserts that two ints are equal. |
| `static void` | `assertEquals(java.lang.String message, long expected, long actual)`             Asserts that two longs are equal. |
| `static void` | `assertEquals(java.lang.String message, java.lang.Object expected, java.lang.Object actual)`             Asserts that two objects are equal. |
| `static void` | `assertEquals(java.lang.String message, short expected, short actual)`             Asserts that two shorts are equal. |
| `static void` | `assertEquals(java.lang.String expected, java.lang.String actual)`             Asserts that two Strings are equal. |
| `static void` | `assertEquals(java.lang.String message, java.lang.String expected, java.lang.String actual)`             Asserts that two Strings are equal. |
| `static void` | `assertFalse(boolean condition)`             Asserts that a condition is false. |
| `static void` | `assertFalse(java.lang.String message, boolean condition)`             Asserts that a condition is false. |
| `static void` | `assertNotNull(java.lang.Object object)`             Asserts that an object isn't null. |
| `static void` | `assertNotNull(java.lang.String message, java.lang.Object object)`             Asserts that an object isn't null. |
| `static void` | `assertNotSame(java.lang.Object expected, java.lang.Object actual)`             Asserts that two objects refer to the same object. |
| `static void` | `assertNotSame(java.lang.String message, java.lang.Object expected, java.lang.Object actual)`             Asserts that two objects refer to the same object. |
| `static void` | `assertNull(java.lang.Object object)`             Asserts that an object is null. |
| `static void` | `assertNull(java.lang.String message, java.lang.Object object)`             Asserts that an object is null. |
| `static void` | `assertSame(java.lang.Object expected, java.lang.Object actual)`             Asserts that two objects refer to the same object. |
| `static void` | `assertSame(java.lang.String message, java.lang.Object expected, java.lang.Object actual)`             Asserts that two objects refer to the same object. |
| `static void` | `assertTrue(boolean condition)`             Asserts that a condition is true. |
| `static void` | `assertTrue(java.lang.String message, boolean condition)`             Asserts that a condition is true. |
| `static void` | `fail()`             Fails a test with no message. |
| `static void` | `fail(java.lang.String message)`             Fails a test with the given message. |

**Methods inherited from class org.testng.internal.junit.ArrayAsserts**

`assertArrayEquals, assertArrayEquals, assertArrayEquals, assertArrayEquals, assertArrayEquals, assertArrayEquals, assertArrayEquals, assertArrayEquals, assertArrayEquals, assertArrayEquals, assertArrayEquals, assertArrayEquals, assertArrayEquals, assertArrayEquals, assertArrayEquals, assertArrayEquals`

**Methods inherited from class java.lang.Object**

`clone, equals, finalize, getClass, hashCode, notify, notifyAll, toString, wait, wait, wait`

**Constructor Detail**

### AssertJUnit

```
protected AssertJUnit()
```

:   Protect constructor since it is a static only class


**Method Detail**

### assertTrue

```
public static void assertTrue(java.lang.String message,
                              boolean condition)
```

:   Asserts that a condition is true. If it isn't it throws
    an AssertionFailedError with the given message.

---


### assertTrue

```
public static void assertTrue(boolean condition)
```

:   Asserts that a condition is true. If it isn't it throws
    an AssertionFailedError.

---


### assertFalse

```
public static void assertFalse(java.lang.String message,
                               boolean condition)
```

:   Asserts that a condition is false. If it isn't it throws
    an AssertionFailedError with the given message.

---


### assertFalse

```
public static void assertFalse(boolean condition)
```

:   Asserts that a condition is false. If it isn't it throws
    an AssertionFailedError.

---


### fail

```
public static void fail(java.lang.String message)
```

:   Fails a test with the given message.

---


### fail

```
public static void fail()
```

:   Fails a test with no message.

---


### assertEquals

```
public static void assertEquals(java.lang.String message,
                                java.lang.Object expected,
                                java.lang.Object actual)
```

:   Asserts that two objects are equal. If they are not
    an AssertionFailedError is thrown with the given message.

---


### assertEquals

```
public static void assertEquals(java.lang.Object expected,
                                java.lang.Object actual)
```

:   Asserts that two objects are equal. If they are not
    an AssertionFailedError is thrown.

---


### assertEquals

```
public static void assertEquals(java.lang.String message,
                                java.lang.String expected,
                                java.lang.String actual)
```

:   Asserts that two Strings are equal.

---


### assertEquals

```
public static void assertEquals(java.lang.String expected,
                                java.lang.String actual)
```

:   Asserts that two Strings are equal.

---


### assertEquals

```
public static void assertEquals(java.lang.String message,
                                double expected,
                                double actual,
                                double delta)
```

:   Asserts that two doubles are equal concerning a delta. If they are not
    an AssertionFailedError is thrown with the given message. If the expected
    value is infinity then the delta value is ignored.

---


### assertEquals

```
public static void assertEquals(double expected,
                                double actual,
                                double delta)
```

:   Asserts that two doubles are equal concerning a delta. If the expected
    value is infinity then the delta value is ignored.

---


### assertEquals

```
public static void assertEquals(java.lang.String message,
                                float expected,
                                float actual,
                                float delta)
```

:   Asserts that two floats are equal concerning a delta. If they are not
    an AssertionFailedError is thrown with the given message. If the expected
    value is infinity then the delta value is ignored.

---


### assertEquals

```
public static void assertEquals(float expected,
                                float actual,
                                float delta)
```

:   Asserts that two floats are equal concerning a delta. If the expected
    value is infinity then the delta value is ignored.

---


### assertEquals

```
public static void assertEquals(java.lang.String message,
                                long expected,
                                long actual)
```

:   Asserts that two longs are equal. If they are not
    an AssertionFailedError is thrown with the given message.

---


### assertEquals

```
public static void assertEquals(long expected,
                                long actual)
```

:   Asserts that two longs are equal.

---


### assertEquals

```
public static void assertEquals(java.lang.String message,
                                boolean expected,
                                boolean actual)
```

:   Asserts that two booleans are equal. If they are not
    an AssertionFailedError is thrown with the given message.

---


### assertEquals

```
public static void assertEquals(boolean expected,
                                boolean actual)
```

:   Asserts that two booleans are equal.

---


### assertEquals

```
public static void assertEquals(java.lang.String message,
                                byte expected,
                                byte actual)
```

:   Asserts that two bytes are equal. If they are not
    an AssertionFailedError is thrown with the given message.

---


### assertEquals

```
public static void assertEquals(byte expected,
                                byte actual)
```

:   Asserts that two bytes are equal.

---


### assertEquals

```
public static void assertEquals(java.lang.String message,
                                char expected,
                                char actual)
```

:   Asserts that two chars are equal. If they are not
    an AssertionFailedError is thrown with the given message.

---


### assertEquals

```
public static void assertEquals(char expected,
                                char actual)
```

:   Asserts that two chars are equal.

---


### assertEquals

```
public static void assertEquals(java.lang.String message,
                                short expected,
                                short actual)
```

:   Asserts that two shorts are equal. If they are not
    an AssertionFailedError is thrown with the given message.

---


### assertEquals

```
public static void assertEquals(short expected,
                                short actual)
```

:   Asserts that two shorts are equal.

---


### assertEquals

```
public static void assertEquals(java.lang.String message,
                                int expected,
                                int actual)
```

:   Asserts that two ints are equal. If they are not
    an AssertionFailedError is thrown with the given message.

---


### assertEquals

```
public static void assertEquals(int expected,
                                int actual)
```

:   Asserts that two ints are equal.

---


### assertNotNull

```
public static void assertNotNull(java.lang.Object object)
```

:   Asserts that an object isn't null.

---


### assertNotNull

```
public static void assertNotNull(java.lang.String message,
                                 java.lang.Object object)
```

:   Asserts that an object isn't null. If it is
    an AssertionFailedError is thrown with the given message.

---


### assertNull

```
public static void assertNull(java.lang.Object object)
```

:   Asserts that an object is null.

---


### assertNull

```
public static void assertNull(java.lang.String message,
                              java.lang.Object object)
```

:   Asserts that an object is null. If it is not
    an AssertionFailedError is thrown with the given message.

---


### assertSame

```
public static void assertSame(java.lang.String message,
                              java.lang.Object expected,
                              java.lang.Object actual)
```

:   Asserts that two objects refer to the same object. If they are not
    an AssertionFailedError is thrown with the given message.

---


### assertSame

```
public static void assertSame(java.lang.Object expected,
                              java.lang.Object actual)
```

:   Asserts that two objects refer to the same object. If they are not
    the same an AssertionFailedError is thrown.

---


### assertNotSame

```
public static void assertNotSame(java.lang.String message,
                                 java.lang.Object expected,
                                 java.lang.Object actual)
```

:   Asserts that two objects refer to the same object. If they are not
    an AssertionFailedError is thrown with the given message.

---


### assertNotSame

```
public static void assertNotSame(java.lang.Object expected,
                                 java.lang.Object actual)
```

:   Asserts that two objects refer to the same object. If they are not
    the same an AssertionFailedError is thrown.

---


### assertEquals

```
public static void assertEquals(byte[] expected,
                                byte[] actual)
```

---


### assertEquals

```
public static void assertEquals(java.lang.String message,
                                byte[] expected,
                                byte[] actual)
```


---


|  |  |  |
| --- | --- | --- |
| **Overview** \| **Package** \| **Class** \| **Tree** \| **Deprecated** \| **Index** \| **Help** | |  |
| **PREV CLASS**   **NEXT CLASS** | **FRAMES**    **NO FRAMES**     **All Classes** |  |
| SUMMARY: NESTED \| FIELD \| CONSTR \| METHOD | DETAIL: FIELD \| CONSTR \| METHOD |  |


---
